# Dosing forgiveness of oral PrEP for cisgender women remains uncertain

**DOI:** 10.1002/jia2.26496

**Published:** 2025-05-19

**Authors:** Mia Moore, David Glidden, Peter Anderson, Craig Hendrix, Dobromir Dimitrov

**Affiliations:** ^1^ Vaccine and Infectious Disease Division Fred Hutchinson Cancer Center Seattle Washington USA; ^2^ School of Medicine University of California San Francisco California USA; ^3^ Skaggs School of Pharmacy and Pharmaceutical Sciences University of Colorado Aurora Colorado USA; ^4^ Department of Medicine Johns Hopkins School of Medicine Baltimore Maryland USA; ^5^ Department of Applied Mathematics University of Washington Seattle Washington USA

1

Pre‐exposure prophylaxis (PrEP) with tenofovir disoproxil fumarate and emtricitabine (TDF/FTC) has been proven safe and effective for preventing HIV acquisition, when taken daily, in men and transgender women who have sex with men (MSM/TGW) and cisgender women (hereafter, women). Based on existing evidence, we can have high confidence that as few as 4 pills per week reduce HIV incidence by at least 90% in MSM/TGW. In addition, a “2‐1‐1” regimen in which two pills are taken prior to a potential HIV exposure followed by one pill in each of the two following days has been clinically proven to substantially reduce HIV incidence in MSM/TGW. However, the same level of support is not yet available for either dosing forgiveness or the efficacy of “2‐1‐1” event‐driven PrEP in women.

One of the most controversial notions in HIV therapeutics is whether women require different adherence or dosing strategies compared with MSM/TGW by virtue of differences in drug distribution between the female genital tract and rectal tissue [1, 2]. The TDF/FTC adherence‐efficacy curve has previously been established in MSM/TGW using levels of intraerythrocytic tenofovir‐diphosphate (TFV‐DP) in incident cases of HIV and matched controls from iPrEx, iPrEx OLE and recently reinforced with HPTN 083 [3, 4, 5].

Four secondary analyses have assessed the relationship between TDF/FTC adherence and HIV incidence in women [5, 6, 7, 8]. These analyses have prompted renewed discussion on the dosing forgiveness in this population and their potential to benefit from on‐demand PrEP [2, 9, 10]. Here, we outline the methodological differences in the latest studies and discuss potential implications for clinical practice guidelines.

Of the four analyses, three concluded that women need to adhere to daily dosing to achieve a 90% reduction in HIV incidence (Figure 1). Two subgroup analyses of non‐randomized cohorts of PrEP users compared HIV incidence in those with high adherence to low adherence [5, 8]. First, women enrolled in HPTN 084 and MSM/TGW enrolled in HPTN 083 had their adherence assessed with intraerythrocytic TFV‐DP, which quantifies adherence over the prior 1–2 months. HIV incidence in those assessed to take <2, 2–3, 4–6 or 7 pills per week was compared to HIV incidence in those with no quantifiable TFV‐DP [5]. Although the confidence intervals were wide, this analysis suggested that women need to adhere to daily pills to gain the same benefit from PrEP as MSM/TGW taking 2–3 pills per week. Second, a meta‐analysis analysed the adherence of 6296 women enrolled in 11 demonstration projects over 8 years [8]. HIV incidence was calculated in four sub‐populations based on adherence which was assessed by various methods including self‐report and drug concentrations. The reduction in HIV incidence among those taking 4–6 and 7 pills per week compared to those taking <2 was comparable to that of Anderson et al. Third, a modelling study reanalysed plasma tenofovir data from placebo‐controlled efficacy studies to derive a relationship between the frequency of pill taking and PrEP efficacy [6]. Moore et al. imputed intraerythrocytic TFV‐DP concentrations from plasma TFV quantifiability with data from HPTN 082 (which collected both plasma and TFV‐DP in dried blood spots) and then calibrated a dose‐efficacy curve to HIV incidence in the placebo and active arms of VOICE, FEM‐PrEP and Partners PrEP. Study results suggest that daily pill taking is required for heterosexual women to achieve the same PrEP benefit as MSM/TGW taking 4 pills per week.

**Figure 1:**
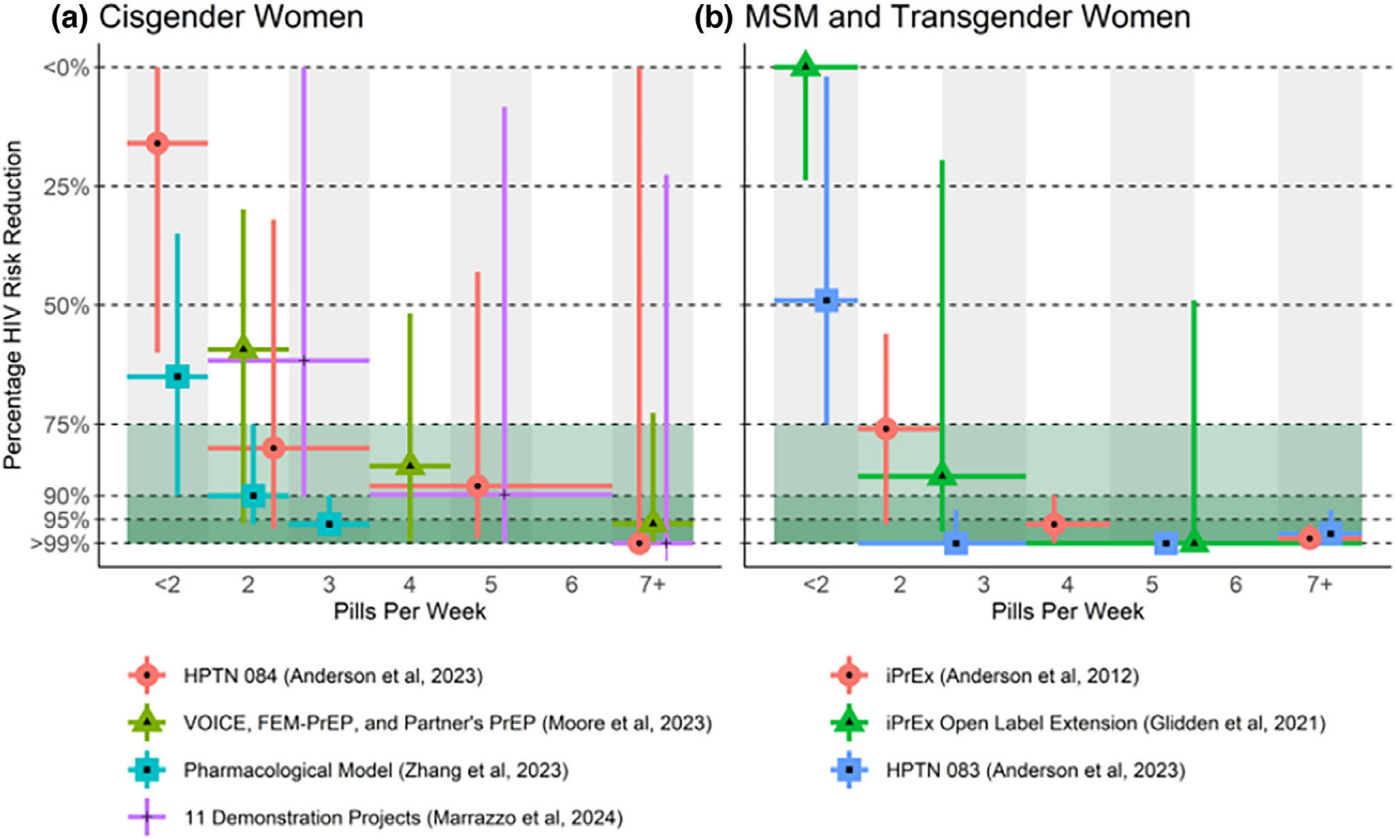
PrEP efficacy by dose. Estimates of HIV risk reduction by number of TDF/FTC tablets taken per week. Vertical bars represent confidence interval of estimate. Horizontal bars represent the range of adherence associated with the estimate. Symbol placement is for readability only. (A) Studies of cisgender heterosexual women. (B) Studies of men and transgender women who have sex with men.

In contrast, Zhang et al. adapted a pharmacokinetic‐pharmacodynamic model of TDF/FTC, previously calibrated to in‐vitro antiretroviral assays, to predict PrEP efficacy based on projected concentrations of active drug metabolites in tissue compartments [7]. They tested hypotheses that intracellular concentrations in either peripheral blood mononuclear cells or local tissues completely determine TDF/FTC efficacy across a range of TDF/FTC doses per week, from one to seven. Assuming each hypothesis in turn and a uniform distribution of weekly pill taking among adherent study participants, they projected the expected reduction in HIV incidence among those with quantifiable plasma tenofovir in the VOICE, FEM‐PrEP, Partners PrEP, HPTN 084 and the TDF2 studies. They concluded that a model in which systemic but not local PrEP concentrations determine PrEP efficacy is consistent with the empiric data. Under this model, PrEP efficacy is expected to be very high in women taking only 1−3 pills per week (Figure 1).

This optimistic result is at odds with the other three studies but has two caveats. First, it is likely that a model in which both systemic and local tissue drug concentrations contribute to PrEP efficacy would fit the data better, as the projections from the two compartments over and underestimated empiric efficacy, respectively. A similar in‐silico model of on‐demand TDF/FTC using drug concentrations in the female genital tract, the more conservative assumption, found that the standard 2‐1‐1 schedule should be extended for cisgender women [11]. Second, this analysis assumed consistent regular pill taking, even among those with non‐daily adherence, which may not reflect real‐life patterns. The 2‐1‐1 dose timing highlights the importance of the pill‐taking pattern and its impact on dosing forgiveness.

In aggregate, dosing forgiveness is evident for cisgender women, but its magnitude and certainty are not as established as for MSM/TGW. Additional data from trials offering daily PrEP as an active control, such as PURPOSE 1, may help inform the dose‐efficacy relationship for women if enough participants use daily PrEP [12]. Furthermore, event‐driven PrEP effectiveness has not been studied in women, and pharmacodynamic model‐based projections rely on data from in‐vitro assays [1, 11]. Non‐daily regimens can be evaluated against daily PrEP using a non‐inferiority cross‐over study design with a counterfactual placebo derived from recency assays [13]. Given the low adherence of women to daily oral PrEP in many studies, it may be necessary to conduct these efficacy trials in sub‐populations with higher adherence such as those in sero‐discordant partnerships. Non‐daily, on‐demand PrEP may be an option for women who face barriers to daily pill taking. Indeed, data from implementation studies show that most women who use PrEP do not adhere to daily pills, highlighting the need for further assessment of oral PrEP efficacy with partial adherence.

## COMPETING INTERESTS

CH has received support for clinical research from Merck and Gilead; in addition, he has licensed patents related to topical microbicides and founded Prionde Biopharma, LLC, a topical microbicide company. PA, DD and MM receive research contracts with Gilead paid to their respective institutions. DG has consulted for Gilead.

## AUTHORS’ CONTRIBUTIONS

MM, PA and DD conceived of the project. MM wrote the initial draft and prepared the figure. All authors revised the manuscript. DD supervised the project.

## FUNDING

This work was supported by two grants from the National Institutes of Health (NIH): 5UM1AI068617‐20, through the National Institute of Infectious Disease (NIAID) and National Institute on Drug Abuse (NIDA); and 1R01AI179417‐01A1 and R01 AI170298 through NIAID.

## Data Availability

Data sharing is not applicable to this article as no datasets were generated or analysed during the current study.
